# Luciferase unleashed: Lighting the path to vaccine approval using a novel high-throughput chikungunya virus neutralization assay

**DOI:** 10.1371/journal.pntd.0014610

**Published:** 2026-08-03

**Authors:** Jason Mendy, Lisa Bedell, Lauren C. Tindale, Jason S. Richardson, Na Li, Yari Fontebasso, Theodore C. Pierson, Keith Gottlieb, Lo Vang

**Affiliations:** 1 Bavarian Nordic Inc., Durham, North Carolina, United States of America; 2 Bavarian Nordic Canada Inc., Toronto, Ontario, Canada; 3 Emergent BioSolutions Inc., Gaithersburg, Maryland, United States of America; 4 Vaccine Research Center, National Institute of Allergy and Infectious Diseases, National Institutes of Health, Bethesda, Maryland, United States of America; 5 Elpida Therapeutics, Encino, California, United States of America; Linköping University: Linkopings universitet, SWEDEN

## Abstract

Chikungunya virus (CHIKV) neutralization assays, which measure virus-neutralizing activity, play a pivotal role in evaluating vaccine effectiveness and monitoring immunity. As CHIKV continues to spread beyond endemic tropical regions, causing more frequent outbreaks and debilitating disease, it remains a serious global health concern. Vaccination can help alleviate these burdens by inducing neutralizing antibodies, which are also produced following natural infection. Neutralizing antibodies can prevent virus infection and are considered a key correlate of protection against disease caused by CHIKV. Accurate assessment of neutralizing antibodies is therefore essential for both vaccine evaluation and public health surveillance. This work introduces a validated luciferase-based high-throughput CHIKV neutralization assay (CHIKV-luc NT_80_) for quantifying serum neutralizing antibodies. The assay uses a genetically engineered reporter virus (CHIKV181/25-luc) that expresses luciferase, allowing *in vitro* viral infectivity to be measured using luminescence rather than manual plaque counting. Compared to the traditional plaque reduction neutralization test (PRNT), this method is faster, easier, more reproducible, and better suited for large-scale clinical studies associated with a high biological sample burden, due to its scalability. The use of a stringent 80% neutralization titer threshold (NT_80_) also provides a more stringent measure of antibodies compared to conventional NT_50_ values. The assay was compared to other methods, having high concordance (r ≈ 0.9) and low bias (≤15%), supporting its analytical reliability, qualified for phase 2 clinical trials, and validated for phase 3 clinical sample testing. The assay contributed to the establishment of the first World Health Organization International Standard for anti-CHIKV (code number 1502/19), and its results harmonized well with other virus neutralization methods. Neutralization titer results from preclinical and clinical serum sample testing supported the characterization of a surrogate threshold of protection, and licensure of VIMKUNYA (CHIKV virus-like particle [VLP] vaccine).

## Author summary

Chikungunya virus (CHIKV), a pathogenic alphavirus, has rapidly emerged as a global health threat, causing widespread outbreaks of debilitating disease and significantly affecting quality of life. Protection from CHIKV disease is possible through vaccination, which can reduce outbreaks, lower healthcare costs, and protect travelers, healthcare workers, and residents of affected regions. Traditional methods for measuring vaccine-induced immunity, such as the plaque reduction neutralization test (PRNT), are relatively slow, labor-intensive, and variable, limiting their scalability and throughput. To address these challenges, a luciferase-based CHIKV neutralization assay (CHIKV-luc NT_80_ assay) was developed, providing a fast, efficient, sensitive, and reproducible readout, making it well-suited for large studies and clinical trials. Using this platform, virus infection is measured by enumerating luciferase activity, whereas the neutralizing activity of serum samples results in a measurable reduction of luminescence. The assay provides a quantitative measure of antibody potency using a rigorous NT_80_ threshold that correlates better with protection. The CHIKV-luc assay was established using existing methods as benchmarks, including PRNT. This assay was validated and used to set a seroprotective threshold and supported clinical trials for the VIMKUNYA vaccine. Additionally, the assay helped establish the first International Standard for chikungunya antibody testing, harmonizing well with other neutralization methods. This new assay supports vaccine research and can help public health efforts to monitor and control the spread of chikungunya.

## 1. Introduction

Chikungunya virus (CHIKV) is an alphavirus transmitted to humans by *Aedes* mosquitoes. Climate change, globalization, and international travel are driving increased global spread of CHIKV disease [1,2]. While rarely fatal, infection from CHIKV can cause fever, rash, and severe joint pain. Over 40% of patients experience chronic symptoms [3,4], causing substantial health and economic burdens [5,6]. The CHIKV virion contains a positive-sense single-strand RNA genome with a long open reading frame coding for capsid (C) and envelope (E1, E2, E3, and 6K) structural proteins, together with 4 nonstructural proteins (nsP1, nsP2, nsP3, and nsP4) required for replication of the virus. The E1 glycoprotein mediates membrane fusion during virus infection of host cells and the E2 transmembrane glycoprotein is responsible for receptor binding to host cells during viral replication [7] and is the primary target of CHIKV neutralizing antibodies induced during natural infection [8]. Notably, protection against subsequent infection has been shown to correlate with the presence of CHIKV antibodies that neutralize the virus *in vitro* [9].

Laboratory tests for CHIKV include a variety of molecular assays such as RT-PCR for detecting viral RNA during acute infection, serological assays like ELISA for identifying IgM and IgG antibodies, and functional assays that measure neutralizing antibodies. Neutralization assays are especially important as they directly assess the ability of antibodies to block viral entry into host cells and other stages of the virus life cycle such as membrane fusion, uncoating, and cell-to-cell spread, providing a key indicator of protection. These tests help determine antibody levels associated with clinically beneficial immunity, guiding regulatory decisions and supporting vaccine approval.

Traditional plaque reduction neutralization tests (PRNT) are considered the gold standard for measuring neutralizing antibodies to CHIKV; however, the method is time-consuming, technically challenging, prone to higher variability, and not ideal for high throughput testing needed in large-scale studies [10]. Viruses used in PRNT assays are usually isolates of circulating strains that require biosafety level 3 (BSL-3) containment. Alternative methods such as the focus reduction neutralization test (FRNT) increase throughput by using 96-well plate formats, often referred to as “micro” neutralization assays, yet still use natural virus. Other microneutralization methods employ pseudo virus, chimaera, recombinant luciferase or green fluorescent protein (GFP) reporter virus systems that are amenable to automation, scalable, and often safer, only requiring BSL-2 containment [11,12]. These features are better suited for large-scale clinical trials associated with a high biological sample burden and collaborative studies where assay standardization, throughput, and scalability are critical.

Two vaccines are currently approved in parts of the world for the prevention of CHIKV disease: a live-attenuated (VLA-1553, IXCHIQ, Valneva) and a virus-like particle (CHIKV VLP vaccine, VIMKUNYA, Bavarian Nordic). VLA-1553 is a single-dose vaccine based on a genetically modified infectious clone of CHIKV strain LR2006-OPY1 (East/Central/South African [ECSA] genotype) isolated during the 2006 La Reunion outbreak [13,14]. CHIKV VLP vaccine is a single-dose vaccine approved for use in individuals 12 years of age and older; the VLP is composed of the CHIKV structural proteins, capsid, envelope 1 (E1), and envelope 2 (E2), derived from the West African Senegal strain 37997 [15]. VLP vaccines are generally considered to offer strong immunity, even after a single dose, and to have a favorable safety profile as they lack replicable genetic material and are therefore unable to infect cells [16,17]. Live-attenuated vaccines are known to offer robust immunity, but have the potential for reversion to virulence, particularly in immunocompromised individuals [18]. Both VLA-1553 and CHIKV VLP vaccine have demonstrated strong immunogenicity, with high levels of neutralizing antibodies observed by 1-month postvaccination for VLA-1553 [13] and by 2 weeks for CHIKV VLP vaccine [19,20].

To support CHIKV VLP vaccine development, a luciferase-based CHIKV neutralization assay (CHIKV-luc NT_80_) was developed. Unlike traditional plaque counting, this assay quantifies reductions in luciferase activity as a measure of viral inhibition, enabling automation and high-throughput testing in a 96-well microplate format, thereby enhancing throughput, reproducibility, and scalability while simplifying operator use. A luciferase reporter virus system was chosen for its high sensitivity and precision, as well as its prior use in other vaccine development studies [21,22]. The CHIKV-luc reporter virus was constructed from a molecular clone of the CHIKV strain 181/25, a live-attenuated derivative of the Southeast Asian isolate AF15561 [23,24]. The CHIKV-luc reporter is heterologous to CHIKV VLP vaccine, which is based on the West African strain 37997. Although CHIKV comprises 4 major lineages (West African, Asian, ECSA, and the Indian Ocean lineage) [25], evidence from NHP studies and clinical trials suggests that CHIKV is a single serotype and that the CHIKV VLP vaccine induces comparable titers of neutralizing activity across multiple CHIKV lineages [15,26].

Although CHIKV exhibits genetic diversity across the West African, Asian, and ECSA/ Indian Ocean lineages, the major neutralizing epitopes on the E1-E2 complex are conserved, and infection or vaccination generally elicits broadly cross‑reactive neutralizing antibodies [15,26,27]. While quantitative lineage-associated differences in neutralization titers have been reported [28], these do not constitute distinct serotypes, as cross-neutralization is maintained. Accordingly, a single representative strain is generally sufficient for immunogenicity assessment in vaccine studies [26].

Given the diversity of serological assays and the lack of standardization in neutralization endpoints, comparisons across studies have limitations. Although a World Health Organization (WHO) International Standard for measuring CHIKV neutralization now exists, it has not yet been widely adopted to harmonize interlaboratory data. This report describes the performance of Bavarian Nordic’s CHIKV-luc NT_80_ assay, highlights differences between the NT_50_ and NT_80_ endpoints commonly used in neutralization assays, and compares this platform with other neutralization technologies, including those evaluated in a WHO collaborative study to characterize the first IS for CHIKV [29]. We also show assay comparison to other neutralization assays, including PRNT, and validation of this novel high-throughput CHIKV-luc NT_80_ assay.

## 2. Methods

### 2.1. Ethics statement

De-identified clinical serum samples were obtained from prior clinical trials evaluating candidate CHIKV vaccines. The samples were collected at multiple clinical trial sites and under study protocols approved by the Walter Reed Army Institute of Research Institutional Review Boards (IRB). Participants had originally provided written informed consent acknowledging their participation and understanding of the intended use of samples for future exploratory research purposes. The clinical studies and execution of serum sample collection, processing, and storage adhered to good clinical practice (GCP) and good clinical laboratory practice (GCLP) guidelines and were conducted in compliance with regulatory requirements. Chikungunya-antibody positive serum samples obtained from epidemiological studies were from participants who were naturally exposed to CHIKV; written informed consent was obtained from participants ≥18 years old and from parents of participants <18 years old (written assent was obtained from children ≥12 years old), and the protocol was reviewed and approved by IRBs of the University of California, Berkeley, the University of Michigan, and the Nicaraguan Ministry of Health.

### 2.2. Assay development

A CHIKV luciferase-based virus (CHIKV-luc) serum neutralizing antibody assay was developed and used to evaluate the immunogenicity of the CHIKV VLP vaccine. The assay was initially used in preclinical studies to measure serum neutralizing NT_80_ antibody titers in mice, rabbits, and rats, and was subsequently qualified and validated for testing nonhuman primate (NHP) serum samples. The assay was used to test sera from a phase 1 clinical trial [30], and these data were compared to other neutralization assay methods, including the PRNT. The CHIKV-luc reporter assay was also qualified to support phase 2 clinical trial sample testing [31–33], validated for phase 3 clinical trials [19,20], and will be further utilized in ongoing and planned clinical trials (NCT06007183, NCT07003984) to evaluate durability and efficacy in real-world settings.

### 2.3. Serum samples

Samples used for testing included sera from the Pediatric Dengue Cohort Study (PDCS) conducted in Managua, Nicaragua evaluating seroprevalence of anti-CHIKV antibodies in participants 2–14 years and ≥15 years of age [34]. Vaccine-elicited antibodies were measured in pre- and post-immune sera obtained from adult participants in VRC311, a phase 1 trial evaluating safety and immunogenicity [30], and VRC704, a phase 2 trial, a dose-finding clinical trial to evaluating safety and immunogenicity [31] of an unadjuvanted version of the CHIKV VLP vaccine. These samples were kindly provided by the National Institutes of Health Allergy and Infectious Diseases (NIAID), Vaccine Research Center (VRC) in Bethesda, MD.

Serum samples were heat-inactivated for 45 minutes at 56 °C prior to testing and stored in qualified freezers at ≤ -70 °C. Positive control serum samples exhibiting low, medium, and high NT_80_ titers were generated from participants from the VRC704 trial by first pooling serum to obtain an approximate high-titer of 2500, then diluting with CHIKV-negative serum to obtain a mid-titer of 1000 and a low-titer of 100. The negative control serum used in the validation experiments was human immunoglobulin G (IgG)- depleted serum (Innovative Research). A human serum test panel of 35 samples with negative, low, medium, high, and very high antibody titers was selected from the VRC311 phase 1 and VRC704 phase 2 trial samples to evaluate assay precision. These samples were drawn from trial populations that were previously tested for NT_80_ titers in prequalification experiments. NT_80_ titers had approximately the following distribution: 10 negatives (titer <10), 10 low (titer 10–100), 5 medium (titer 100–1000), 5 high (titer 1000–10,000), and 5 very high (titer >10,000).

### 2.4. Generation of the CHIKV-luc reporter virus

**Fig 1:**
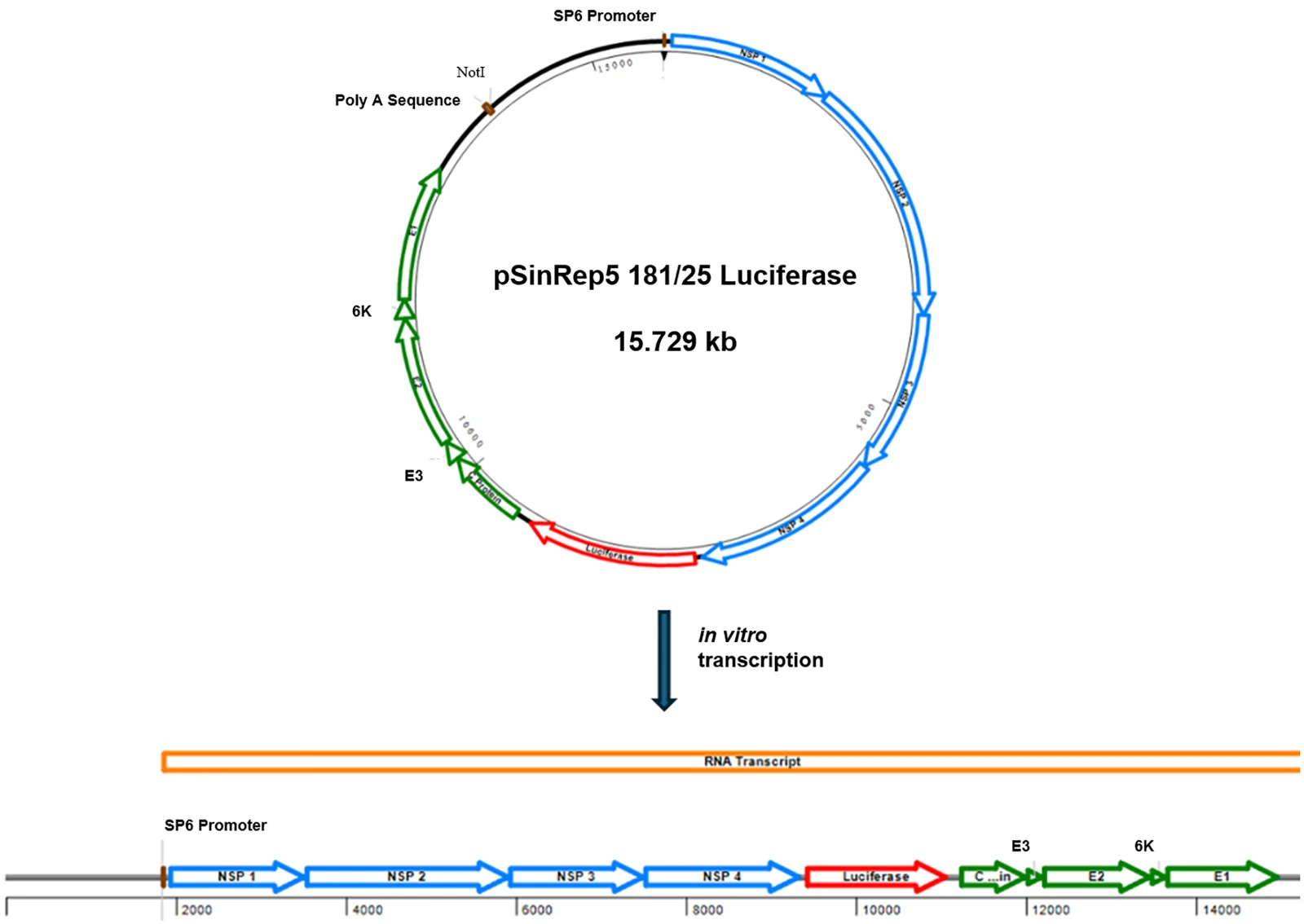
Diagram of the pSinRep5 plasmid and transcribed viral RNA encoding the CHIKV-181/25 luciferase genome. The chikungunya virus-181/25 nonstructural proteins (blue), luciferase (red), and structural polyprotein open reading frame (green) locations are shown within the pSinRep5 DNA plasmid. The luciferase transgene was inserted just downstream of the 26S subgenomic promoter.

The CHIKV-luc reporter virus is a recombinant CHIKV-181/25 virus genetically modified to express firefly luciferase (luc) during replication. CHIKV-181/25 is the live-attenuated derivative of Southeast Asia human isolate strain AF15561 (Gorchakov 2012) and is considered a BSL-2 level virus as discussed in publications from the US Department of Health and Human Services, Centers of Disease Control and Prevention, and Biosafety in Microbiological and Biomedical Laboratories. To generate the virus, a plasmid encoding the full-length cDNA of the CHIKV-181/25 genome (including nonstructural (nsP1-4) and structural genes (C, E3, E2, 6k, and E1)) was obtained from the University of Texas Medical Branch (UTMB). The infectious clone of CHIKV-181/25 was modified to express firefly luciferase under the control of an internal subgenomic (sg) promoter. The luciferase transgene was synthesized and inserted under the control of an sg between nsP4 and the naturally occurring sg promoter, which drives expression of the structural polyproteins, using SwaI and SfiI restriction endonuclease digestion and ligation. Sequencing across the insert was used to confirm correct placement and the absence of mutations. Full-length viral RNAs were synthesized from NotI-linearized plasmid by *in vitro* transcription using the mMESSENGER mMACHINE SP6 Transcription Kit (Ambion, Austin, TX, USA). The 5’ capped RNA was transfected into Vero cells by electroporation, and the cells were incubated for 3 days at 37 °C with 5% CO_2_. Supernatants were collected and characterized. Virus titration on Vero cells and plaque and Steady Glo luciferase assays were performed to confirm the presence and concentration of infectious virus and luciferase expression. The design of the CHIKV-luc molecular clone is shown in Fig 1.

### 2.5. Vero cell culture

Vero cells (CCL-81, ATCC) susceptible to CHIKV infection were obtained from the American Type Culture Collection (Manassas, VA) and used for CHIKV production and plaque and neutralization assays. The cells were propagated in T225 tissue culture flasks with DMEM high-glucose media containing L-glutamine, 10% heat-inactivated (HI) FBS, and 50 µg/mL gentamycin. Cell culture procedures were performed using sterile technique using a biosafety cabinet in a BSL-2 facility, and the cells were incubated in incubators set at 37 °C with 5% CO_2_. The cells were cultured in growth media until 80–90% confluence, then harvested using 0.25% trypsin-EDTA for subculturing or in preparation for virus expansion, plaque assays, or neutralization assays. Vero cells were used for up to 40 passages, after which a new stock of low-passage cells was obtained from a master cell bank stored in liquid nitrogen. Harvested cells were seeded into T225 flasks (Corning, Corning, NY; USA) for further propagation or expansion of the CHIKV-luc virus, 12-well tissue culture plates for plaque assays and PRNT, and 96-well tissue culture plates for the CHIKV-luc NT_80_ assay.

### 2.6. Expansion of CHIKV-luciferase virus

The CHIKV-luc virus was expanded to generate a virus master bank by infecting permissive Vero cell monolayers with RNA transfection lysate. Cell monolayers were established by seeding T225 flasks (Corning, Corning, NY; USA) with 1.25 x 10^6^ Vero cells and incubating for 3 days at 37 °C until the cells reached approximately 70% confluency. Each flask was infected by adding 10 mL of a 1:20 dilution of infectious RNA transfection lysate diluted in growth medium and incubating for 2 hours at 37 °C with 5% CO_2_. After adding additional media, the plates were incubated for 2–3 days until the cytopathic effect reached approximately 80%. The cells and supernatants from each flask were harvested, clarified by centrifugation, and sterile filtered using a 0.2 µm vacuum filter. Pooled clarified virus was aliquoted and stored at ≤-70°C. Plaque assays were used to detect infectivity and determine titer, and the Steady Glo luciferase assay system (Promega, Madison, WI, USA) was used to verify luciferase activity (luminescence signal) using the manufacturer’s instructions.

### 2.7. CHIKV-luc virus titration

Vero cells were seeded at 1.5 x 10^4^ cells/well into each well of 96-well plates and incubated overnight at 37 °C to establish 70–80% confluent monolayers. Serial 2- and 3-fold dilutions of CHIKV-luc virus were prepared in DMEM, ranging from 1:10 to 1:5120 and 1:10 to 196,830, respectively, and then added to Vero cells in 8 replicate wells per dilution. The cells were incubated for 20 hours, and Steady Glo luciferase assays (Promega) were performed according to the manufacturer’s instructions to determine infected wells.

### 2.8. Chikungunya luciferase neutralization assay

**Fig 2:**
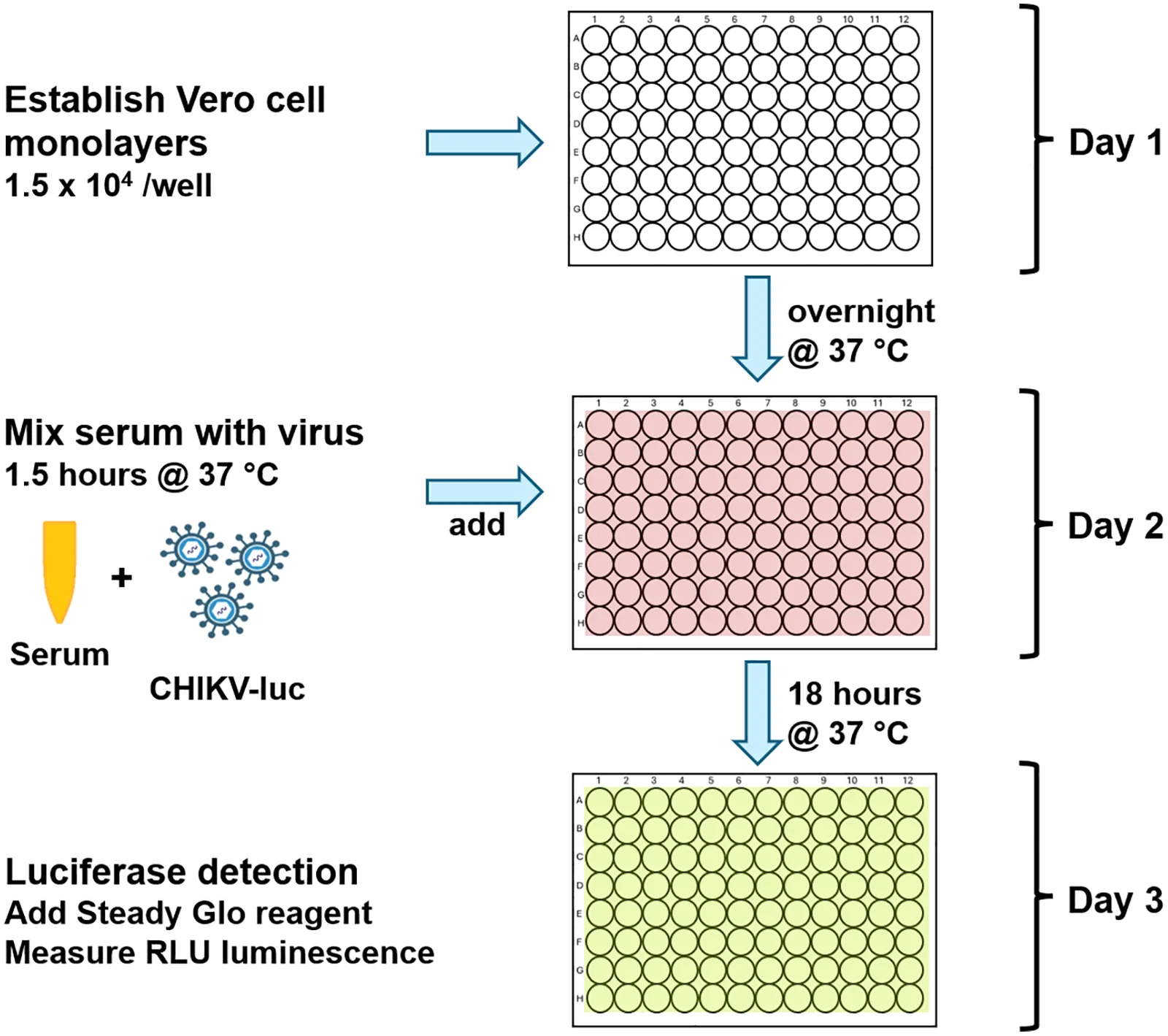
Flow Diagram of the CHIKV-luc NT_80_ Assay. Vero cell monolayers are first established in 96-well culture plates (Day 1). Dilutions of serum are incubated with CHIKV-luc virus and the mixtures added to the Vero cells (Day 2). Luciferase activity is detected by adding luciferin substrate then measuring relative light unit (RLU) luminescence signal on a plate reader luminometer (Day 3).

Assays were conducted in a phase-appropriate manner using pre-established protocols or standard operating procedures and, with qualified or validated equipment, reagents, operators, and electronic systems. Assay plates were prepared by seeding 96-well tissue culture plates (Corning) with 1.5 x 10^4^ Vero cells/well to establish Vero cell monolayers of approximately 80–90% confluency. For neutralization assays, serial 2.5-fold dilutions, starting with a 1:5 dilution of heat-inactivated sera, were prepared and mixed with an equal volume of a fixed concentration of CHIKV-luc reporter virus, and incubated for 90 minutes at 37 °C with 5% CO_2_. Final serum dilutions ranged from 1:10 to 1:15,259. An extended dilution series ranging from 1:156 to 1:238,419 was occasionally used for samples exhibiting neutralization at the last dilution (i.e., there was indication that the titer may be higher than 15,259) to determine titer. Following virus-serum incubation, each serum virus mixture was added in triplicate to designated wells of assay plates containing Vero cells and incubated for 20 hours at 37 °C with 5% CO_2_. Controls included in each assay were sera with known 1) high, 2) medium, and 3) low positive CHIKV neutralizing antibody titers, 4) a negative control with no detectable CHIKV titer, 5) a virus control (VC), and 6) a cell control (CC). The VC included on each assay plate consisted of Vero cells infected with untreated virus to establish the maximum luminescence signal for the assay, representing no antibody neutralization in the assay. The CC had only cells, with no virus or serum added, which generated minimal or no signal, representing 100% neutralization. Luciferase activity was measured using the Steady-Glo Luciferase reagent system (Promega) and relative light unit (RLU) luminescence read on an Envision plate reader luminometer (Perkin Elmer, Shelton, CT). The Steady-Glo Reagent was prepared according to the manufacturer’s specifications and recommendations. To scale the assay for high volume sample testing, automation was incorporated at specific steps in the workflow including a Multidrop Combi reagent dispenser (Thermo Scientific) for seeding the Vero cell 96-well assay plates and dispensing luciferin detection reagent. Serial dilutions of serum samples were automated using Viaflo 96 electronic pipetting systems (Integra Biosciences, Hudson, NH) and plate stackers added to the luminometers for reading up to 44 assay plates per run. The 80% neutralizing antibody titer (NT_80_) was defined as the maximum serum dilution that reduced 80% of luciferase activity compared to virus control wells using linear regression and interpolation. The diagram in Fig 2 shows the workflow performed over a 3-day period.

#### 2.8.1. Calculation of NT_80_.

The NT_80_ antibody titers were determined using linear regression analysis and interpolation. Titration curves were used to establish a local linear relationship between the log_10_-transformed serum dilutions and the luminescence signal over a subsection of the dilution range. Net RLU for each test and control well were calculated by subtracting the average of the CC well values from each RLU of test and control well values. The average net RLU for replicate wells was determined and the percent neutralization was calculated using the formula:


$$\begin{array}{c} \%\text{ Neutralization}=\left[\text{1}-\left(\text{Average }{\text{RLU}}_{\text{Dilution}}÷\text{Average }{\text{RLU}}_{\text{VC}}\right)\right]\times \text{100} \end{array}$$

Average RLU_Dilution_ = background subtracted average RLU of each test dilution

Average RLU_Virus Control_ = background subtracted average RLU of the VC wells

VC = virus control

The NT_80_ representing the highest serum dilution that reduced maximum RLU luminescence signal (corresponding to maximum virus infection and luciferase activity) was determined using linear regression to interpolate the upper- and lower-percent neutralization values that bracket the endpoint (i.e., an interpolated estimate of the dilution that corresponds to 80% neutralization). For development and preclinical studies, calculation of NT_80_ antibody titers was performed using GraphPad Prism (www.graphpad.com). To increase analysis throughput, calculation of NT_80_ titers was automated and performed using a custom R package [35]. The R package was validated for use in the NHP and clinical studies.

#### 2.8.2. NT_80_ vs NT_50_ reporting and analysis.

The NT_50_ and NT_80_ titers corresponded to the highest serum dilution that provided 50% and 80% protection of Vero cells from CHIKV-luc infection, respectively. One way analysis was used to compare the geometric mean ratios of each test run in the collaborative study for each sample and using the variance to determine overall difference between NT_50_ and NT_80_. Bivariate fit analysis was used to compare the geometric mean titers of NT_50_ and NT_80_ across two variables to see if there was a consistent ratio between them and highlight any variability between them. Linear fit, which is a form of linear regression, was used to assess the relationship between NT_50_ and NT_80_ by fitting a straight line to the data points and evaluating whether a proportional relationship existed. Precision analysis was used to observe variations of repeatability or how closely the repeated test results (titers) agreed with each other and variation was calculated as coefficient of variation (%CV).

### 2.9. Plaque assay

Plaque assays were used to detect and quantify infectious viruses in transfection lysates and supernatants from virus expansion. Vero cells cultured in DMEM high glucose with L-glutamine containing 10% FBS and 50 µg/mL gentamycin were harvested and seeded in duplicate into 12-well high-binding tissue culture plates (Corning) at 2 x 10^5^ cells per well and incubated overnight at 37 °C with 5% CO_2_ to establish monolayers. Serial 1:10 dilutions of virus sample were prepared in DMEM high glucose with L-glutamine supplemented with 2% FBS and 50 µg/mL gentamycin, added to the Vero cells in 12-well plates and incubated for 90 minutes at 37 °C with 5% CO_2_. After adsorption, the cells were washed with PBS and an overlay media consisting of DMEM growth media and 0.9% agarose added to each well. The plates were incubated for 3–4 days until plaques were observed. An additional overlay media containing 0.015% neutral red stain was added to each well, and the plates were incubated for an additional day. Plaques were counted manually by two operators. Plaque-forming units per mL (PFU/mL) were calculated by dividing the number of plaques by the infection volume.

### 2.10. Plaque reduction neutralization test (PRNT_80_)

The CHIKV PRNT_80_ neutralization assay was adapted from methodologies of prior studies [9,30]. Serial 1:2 dilutions of test serum previously heat-inactivated for 45 minutes at 56 °C were mixed 1:1 v/v with a known concentration of CHIKV-181/25 virus for 1 hour at 37 °C. The mixtures were then added to pre-established Vero cell monolayers in 12-well plates and incubated for 1 hour at 37 °C. The monolayers were overlaid with 1% agarose and incubated further for 3 days. A neutral red overlay was added, and plates incubated an additional day. Negative control wells resulted in approximately 60 plaques. Neutralizing antibody titers were expressed as PRNT_80_, the reciprocal of the interpolated serum dilution resulting in 80% reduction of plaque formation.

### 2.11. SFV/CHIKV-GFP neutralization assay

The SFV/CHIKV-GFP neutralization assay used to test serum samples from participants in the VRC311 phase 1 clinical trial was performed as described [30]. The assay used a GFP-expressing Semliki Forest Virus (SFV)/ CHIKV chimeric virus and measured neutralization based on the reduction of GFP-expressing cells in the presence of immune serum. Briefly, serial 1:3 dilutions of heat-inactivated serum (for 45 minutes at 56 °C) were incubated for 1 hour at room temperature with a known concentration of a chimeric SFV/CHIKV-GFP virus that generated a maximum number of GFP-expressing cells. The mixtures were then added to Vero cell monolayers pre-established in 96-well plates at 2.5x10^4^ cells/well, and plates incubated for 8 hours at 37 °C with 7% CO_2_. After this incubation, the cells were permeabilized with trypsin and fixed with paraformaldehyde. GFP-positive infected cells were measured by flow cytometry. Dose-response data were fitted using a four-parameter logistic (4PL) regression model to estimate IC_50_ and IC_80_ titers.

### 2.12. Assay validation

#### 2.12.1. Intra-assay precision.

Intra-assay precision refers to repeatability of the assay under the same conditions usually the variability of replicate testing within the same assay. For the CHIKV-luc NT_80_ assay, a single assay run consisted of 81 samples with unique virus preparation and identical handling, including incubation times. Samples repeatedly tested within this same assay, therefore, were used to evaluate intra-assay precision (repeatability). Intra-assay precision was determined by testing 6 positive anti-CHIKV human serum samples with approximately low (10–100), medium (100–1000), or high (1000–10,000) titers a total of 9 times per assay. A total of 6 assays were performed with 2 technicians each testing 1 of the 6 samples a total of 9 times per assay. A linear mixed model was fitted to all data (log NT_80_) with sample as fixed effect, technician and day as random effects. The residual variance estimated using REML was used to calculate the overall coefficient of variation (CV) percentage. The following formula was used for %CV (where σ is the standard deviation on the natural log scale and σ^2^ is the residual variance). Acceptance criteria: the overall CV for intra-assay precision was less than or equal to 25%.


$$\begin{array}{c} \%CV=\text{1}\text{0}\text{0}\;\times \;\sqrt{e^{\sigma^{\text{2}}}\;-\text{1}} \end{array}$$

#### 2.12.2. Inter-assay precision.

Inter-assay precision or intermediate precision involves measuring variability under changing conditions such assays run on different days and by different operators. For the CHIKV-luc NT_80_ assay, the conditions evaluated included separate days and technicians. Inter-assay precision was determined by testing 35 human serum samples with anti-CHIKV titers across the range of the assay on 4 different days by 2 technicians per day for a total of 8 assays. A linear mixed model was fitted to all data (natural log titer) with sample as fixed effect, technician and day as random effects. Individual variance components were summed to produce the total variance for the calculation of the overall %CV for inter-assay precision using the same formula as above (where σ^2^ is equal to the total variance). Acceptance criteria: the overall CV for inter-assay precision was less than or equal to 35%.

#### 2.12.3. Linearity.

Linearity is the ability to elicit test results that are directly, or by a well-defined mathematical transformation, proportional to the concentration of analyte in samples within a given range. In this analysis, 3 anti-CHIKV human serum samples with a high titer of neutralizing activity were serial 2.5-fold diluted independently in a pool of negative human serum to prepare samples with titers across the range of the assay. The samples were diluted to include at least one dilution at the level expected to be negative. Each of the 3 sets of dilutions (including undiluted) were tested in 2 independent assays, with each assay performed on a different day and by a different technician. A 1^st^ order linear regression analysis using the natural log transformed titers (y-axis) and natural log transformed reciprocal of the sample dilution (x-axis) was performed for each of the 3-sample dilution sets independently. Since the validation aimed to confirm the lower limit of quantitation (LLOQ) value of 15, linearity values <15 were not included in analysis. The undiluted sample was given a dilution factor of 1 and, therefore, equivalent to a natural log transformed value of 0. Acceptance criteria: the 1^st^ order linear regression analysis using the log transformed titer results from the positive titer dilution samples must result in a coefficient of determination >0.95 and a slope with 90% CI within the range of -0.7 to -1.3. Criteria were assessed for each of the 3 samples independently.

#### 2.12.4. Limit of detection.

The limit of detection (LOD) is constrained by the minimum serum dilution value of 10, which is the starting dilution of the assay. The LOD was evaluated from the repeated testing of 10 negative (<10) and 10 low titer (10–100) anti-CHIKV human serum samples from the inter-assay precision parameter. The LOD was defined as the lowest titer at which 95% of repeated observations yielded a reportable value >10.

#### 2.12.5. Lower limit of quantitation.

The LLOQ was determined using the results of the linearity study in combination with the low-titer (10–100) inter-assay precision results. The LLOQ was confirmed as the lowest titer that equals or exceeds the LOD and at which the assay is linear and has acceptable inter-assay precision.

#### 2.12.6. Robustness.

Robustness measures the capacity of the assay to remain unaffected by naturally occurring variation in method parameters. To evaluate robustness, variations of assay parameters were tested including: 1) stability of serum after multiple freeze thaw cycles, 2) stability of serum at different temperatures and storage times, 3) incubation range of the neutralization step, 4) incubation range of the overnight infection, and 5) Steady-Glo incubation time. Stability of serum was evaluated after each of 4 freeze/thaw cycles and compared to a sample that had only undergone one freeze thaw cycle. For each freeze/thaw cycle, a sample was frozen at least 18 hours at <-60 °C and thawed at least 3 hours at room temperature (RT). Samples stored at RT or 2–8 °C for 7, 14, and 28 days were compared to a sample that was removed from <-60 °C storage and tested with approximately 3 hours. Human serum samples with low, medium, and high anti-CHIKV positive titers were tested for each condition. For freeze/thaw and sample stability analyses, a linear mixed model was fitted with natural log (NT_80_), with sample as a fixed effect, and test day (technician effect is confounded with test day) as random effect. For freeze/thaw analysis, the number of freeze/thaw cycles (1–4) was included as discrete variable fixed effect, which allowed the calculation of overall geometric mean ratios (across all samples) between 2–4 cycles versus 1 cycle (the reference condition). For sample stability analysis, the condition (normal, RT, 7, 14, or 28 days at 2–8°C) was included as fixed effect, which allowed the calculation of overall geometric mean ratios (across all samples) of different conditions against normal (the reference condition). For incubation range of neutralization and overnight infection, high, medium, and low positive anti-CHIKV human serum samples were tested at n = 6 each was tested in two separate assays evaluating the high and low end of the incubation range. For Steady-Glo incubation time, 10 positive samples were tested and assay plates read at 20 min, 1, 2, 3, 4, and 5hrs after addition of Stead-Glo substrate. Acceptance criteria: the 90% CI of the ratio of each test condition to the reference condition was between 0.7 to 1.4.

#### 2.12.7. Selectivity.

Selectivity is the ability of an assay to identify a particular analyte in the analyzed matrix without interference from matrix components. Selectivity was evaluated in hemolyzed serum, lipemic serum, and plasma samples. For hemolyzed and lipemic serum, two spiked hemolyzed serum and two lipemic individuals at approximate titer levels 3300 and 15 (LLOQ) were tested n = 3 in a single assay along with the unspiked reference condition. For plasma, 10 spiked plasma individuals at approximate titer levels 3300 and 15 (LLOQ) were tested along with the reference condition (unspiked). Acceptance criteria: the 90% CI for the ratios for a given test condition was within 0.7 and 1.4.

## 3. Results

### 3.1. Generation of the CHIKV-luc reporter virus

The CHIKV-luc virus was produced by *in vitro* transcription of the plasmid cDNA followed by transfection of Vero cells with the RNA to produce the viral proteins that self-assemble into infectious particles. After transfection, the infected Vero cell lysate supernatant was tested for luciferase expression to confirm the presence of infectious virus. By design, the CHIKV-luc reporter virus expressed luciferase after infection and luminescence was produced when exposed to its substrate luciferin. Since the expression of luciferase and the measurement of luminescence are proportional to infectivity, the presence of CHIKV-luc virus in transfection lysates was confirmed by titrating the supernatant on Vero cell monolayers using the Steady Glo luciferase assay. The viral lysate showing the best overall profile of luciferase expression was further expanded by infecting multiple flasks of Vero cells to produce a large virus master bank. Following virus expansion, the CHIKV-luc virus was harvested, pooled, and characterized using both luciferase and plaque assays. The plaque assay resulted in 73 average plaques (74 + 72 plaques)/2 replicates at a 1:10,000 virus dilution. The following equation was used to determine PFU/mL, where n = number of plaques, d = virus dilution, and v = inoculate volume in mL.


$$\begin{array}{c} \text{PFU}/\text{mL}=\text{n}/(\text{d}*\text{v}) \end{array}$$

$$\begin{array}{c} \text{PFU}/\text{mL}=\text{73 plaques}/\left({\text{10}}^{-\text{4}}*\text{0}.\text{26}\right)=\text{2}.\text{81}\times {\text{10}}^{\text{6}} \end{array}$$

**Fig 3:**
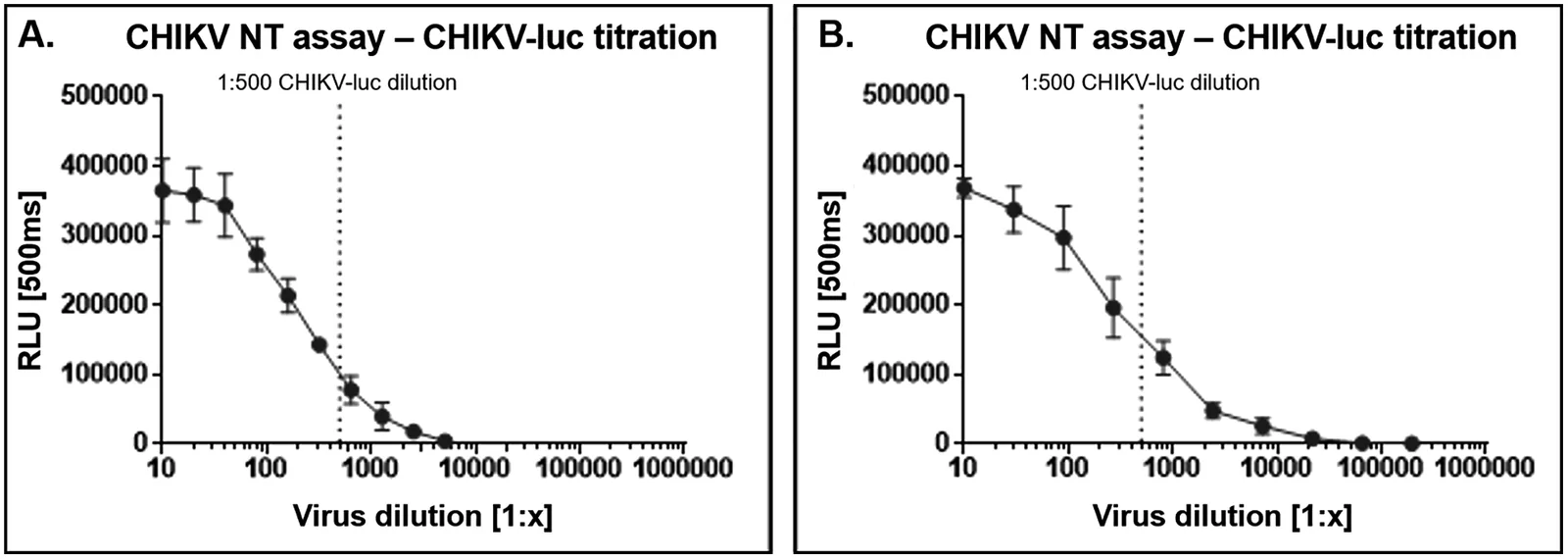
CHIKV-luc virus titration. Virus titration curves of luciferase activity after infecting Vero cells with 2- (panel A) and 3-fold (panel B) dilutions of CHIKV-luc reporter virus. Ten total dilutions were tested for each dilution series.

The master stock of CHIKV-luc reporter virus was further characterized to determine luciferase expression, dynamic range, and the fixed concentration of virus to use in the CHIKV-luc NT_80_ assay. Two- and three-fold serial dilutions were tested, and the titration curves of luciferase activity corresponding to infectivity are shown in Fig 3.

The optimal virus dilution was chosen from a point within the linear part of the virus titration curve to avoid saturation of luciferase activity and obscuring neutralizing activity. Testing replicate titrations of low and high titer CHIKV-positive samples with 1:500, 1:1000, 1:2000, and 1:4000 dilutions of the virus resulted in coefficients of variation (CV) between 10.09% to 32.93% for high titer and 6.97% and 25.12% for low titer samples across the 500- to 4000-fold virus dilution range. The precision of the virus control (VC) RLU also decreased, from 4.19% to 22.21%. A 1:500 dilution of the master stock of virus was initially chosen for the fixed concentration of virus (5.62 x 10^3^ PFU/mL or approximately 281 PFU/well) used in the developed assay although later qualified to be used at a 1:185 dilution (1.52 x 10^4^ PFU/mL or approximately 760 PFU/mL). Bridging qualification of a second lot of virus was used at a 1:240 dilution.

### 3.2. Development of the CHIKV-luc NT_80_ assay

The development of the CHIKV-luc NT_80_ assay followed a structured and iterative path, with each stage refined and executed in a phase-appropriate manner. Optimal assay conditions were determined by systematically testing virus concentration, cell density, media composition, plate layout, edge effect, neutralization time, duration of infection, positive and negative controls, and dilution scheme. Automation incorporated into the assay workflow increased throughput. The assay was further optimized for sensitivity, reproducibility, and scalability. The assay was initially developed to test serum samples from preclinical mouse immunogenicity and epidemiological studies. It was further developed, compared to other methods, supported preclinical passive transfer/challenge, toxicity, and impact on reproduction in mice, rats, and rabbits respectively, vaccine impact on reproductive in rabbits, and qualified and validated human phase 2 and 3 clinical trials.

**Fig 4:**
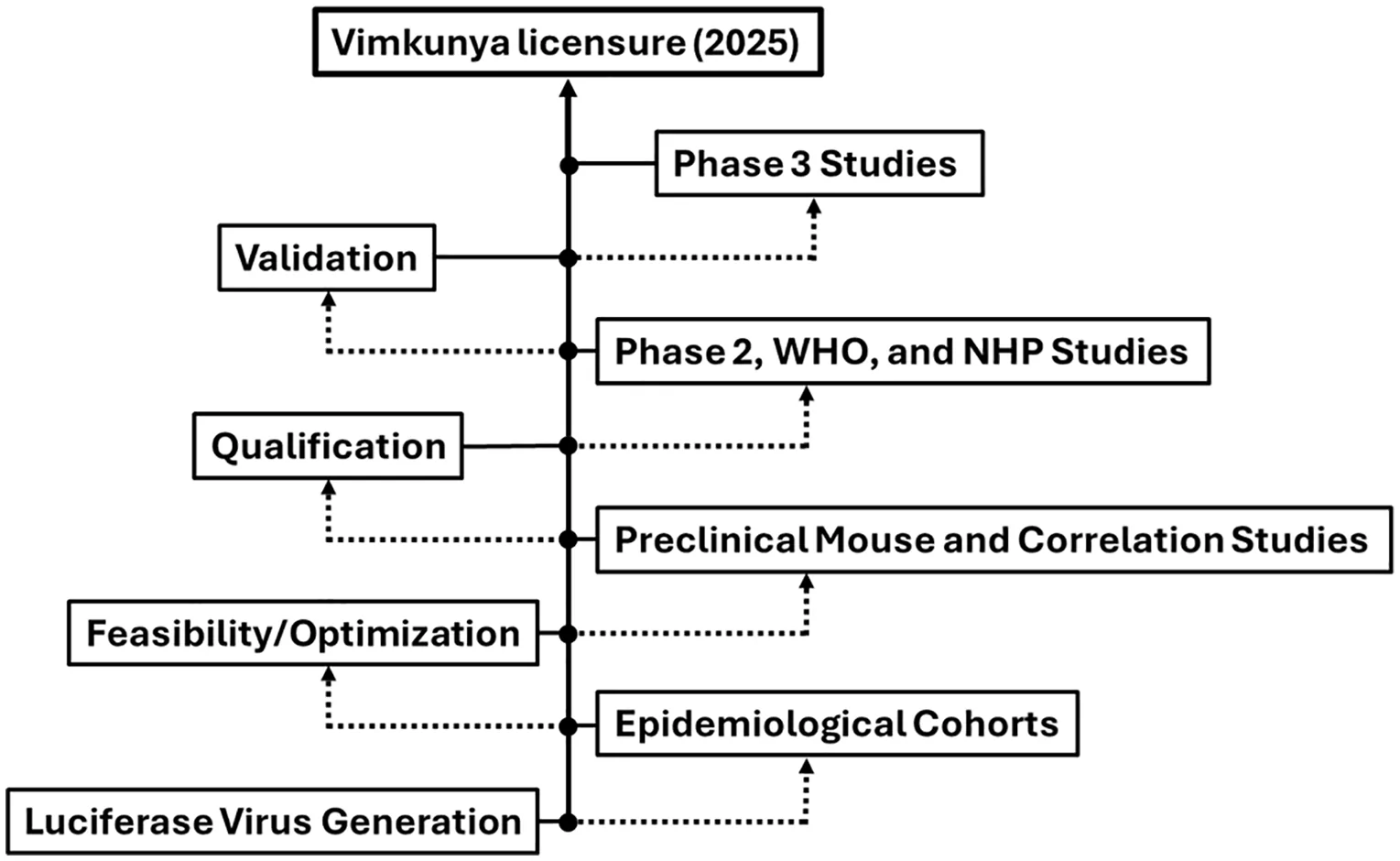
Flow Diagram of Assay and Vaccine Development Pathways. Development of the CHIKV-luc NT_80_ assay started with generation of the luciferase-expressing CHIKV-181/25 reporter virus. The assay was developed using samples collected from epidemiological cohort studies and further optimized to test sera from preclinical and clinical samples. Qualification and validation of the assay enabled testing of phase 2 and 3 clinical serum samples, respectively.

The assay and vaccine development pathways followed a parallel and interconnected path (Fig 4). Testing samples from preclinical animal studies, epidemiological cohorts, and historical clinical trials confirmed its reliability and feasibility for immunogenicity testing. Results from assay correlation studies demonstrated comparability with other methodologies including PRNT and a flow cytometry-based method. The assay was qualified and used to support phase 2 clinical sample testing and was further utilized in a WHO collaborative study to characterize the first chikungunya International Standard. The assay was specifically qualified and validated for testing serum from NHPs supporting active immunization and passive transfer and challenge NHP studies and validated for a pivotal NHP study testing passively transferred serum from humans that received the vaccine dose formulation selected for phase 3 trials and heterologous virus challenge that established a surrogate threshold of protection. To support phase 3 clinical trials and human serum testing, the assay was validated under GCLP guidelines. The development and application of the CHIKV-luc NT_80_ assay represents a significant advancement in the evaluation of viral vaccines, aligning with innovative efforts to modernize and streamline assay readouts. Each milestone in the progression of this assay, from the creation of a stable reporter virus to validation and regulatory approval, demonstrates the increasing precision, efficiency, and practicality that luciferase-based neutralization assays provide for vaccine development.

### 3.3. Correlation with other methods

To aid in the development of the CHIKV-luc NT_80_ assay and compare neutralizing antibody responses determined during a previously completed clinical trial [30], retrospective testing of archived serum samples was performed. This post hoc analysis was not part of the original trial protocol and did not directly support predefined clinical endpoints. Assay correlation studies were conducted using incurred samples from the VRC311 phase 1 clinical trial. The samples were tested to demonstrate that the CHIKV-luc NT_80_ assay produces comparable results to 1) a traditional PRNT assay, and 2) the GFP flow cytometry-based method used by the NIAID to support the immunogenicity endpoints of the VRC311 trial. The PRNT assay was developed based on the methodology used in the AFRIMS Philippine field trial that established a link between CHIKV neutralizing antibodies and protection [9]. Each virus neutralization method is summarized in Table 1.

**Table 1:** Summary of the CHIKV neutralization assays.

Feature	PRNT	SFV/CHIKV	CHIKV-luc
Source	AFRIMS	NIAID	Bavarian Nordic^1^
Use	Philippine cohort trial	VRC311 phase 1 trial	Preclinical/clinical studies
Assay type	Plaque reduction	Flow cytometry	Microneutralization
Virus used	CHIKV-181/25	SFV/CHIKV chimera	CHIKV-181/25
Lineage	Asian	Indian Ocean	Asian
Strain	AF15561	LR2006 OPY1	AF15561
Detection	Plaque counting	GFP expression	Luciferase activity
Reported titer	PRNT_80_	IC_50_, IC_80_^2^	NT_80_
Throughput	Low	Medium	High
Biosafety level	BSL-3	BSL-3	BSL-2

^1^As successor in interest to Emergent BioSolutions Inc. and formerly PaxVax.

^2^IC_80_ titers were calculated from the original neutralization data generated during the VRC311 trial.

Serum samples (n = 146) from 25 participants who had received escalating doses (10, 20, or 40 ug) of the CHKVLP059 vaccine at several time points (weeks 0, 4, 8, 24, and 44) in the VRC311 trial were run in each assay and three pairwise comparisons were performed analyzing the data side by side as follows: 1) PRNT_80_ vs. SFV/CHIKV OPY1 IC_80_, 2) CHIKV-luc NT_80_ vs. SFV/CHIKV OPY1 IC_80_, and 3) PRNT_80_ vs. CHIKV-luc NT_80_.

For a quantitative assessment of correlation and bias between each pair of assays, only samples with quantifiable titer were included in the analysis. Each pair was evaluated by measuring the nonparametric Spearman correlation coefficient, a measure of the strength of association between each pair, and median bias, or the median percent difference of reportable titer. A 95% confidence interval using binomial theory was used for bias.

**Fig 5:**
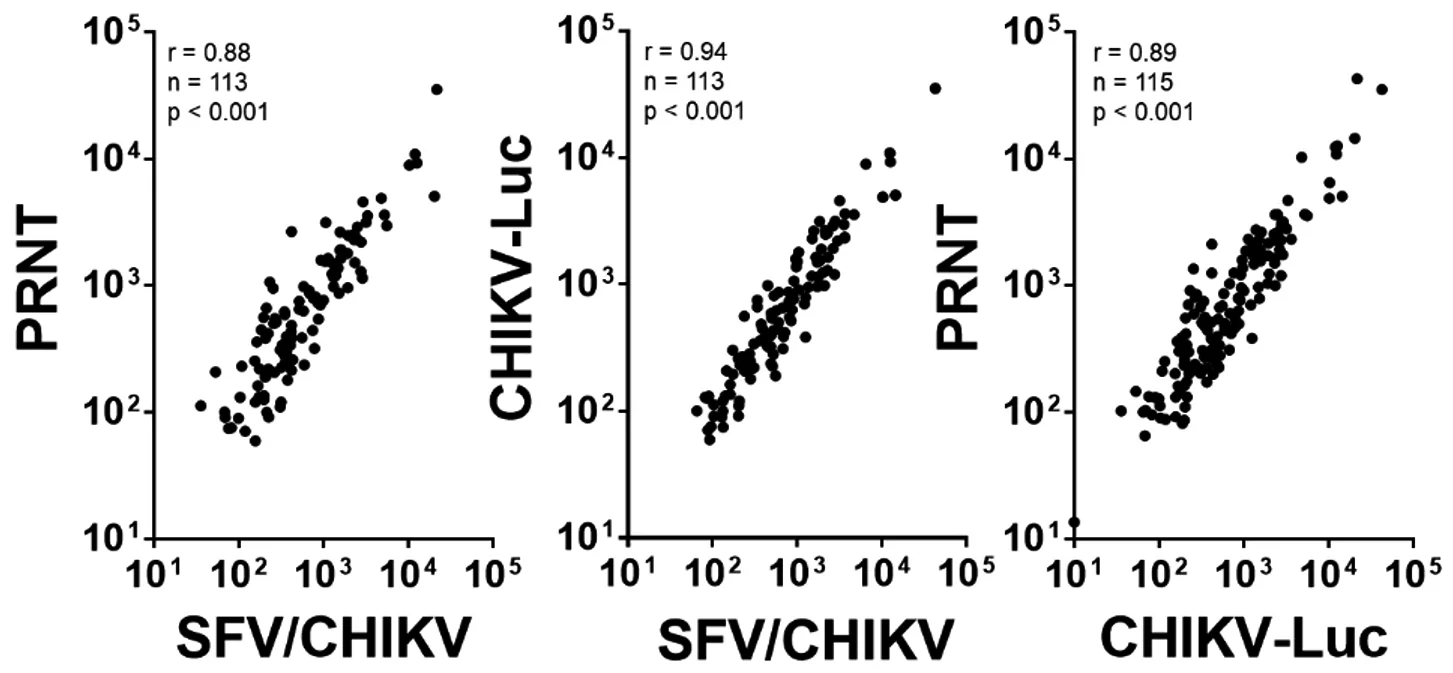
Pairwise comparison of CHIKV-specific antibody titers from the PRNT_80_, SFV/CHIKV OPY1 IC_80_, and CHIKV-luc NT_80_ assays. Scatter plots of each of three pairwise assay comparisons of data generated from testing VRC311 samples are shown, including 1) PRNT_80_ versus SFV/CHIKV IC_80_ (left panel), 2) CHIKV-luc NT_80_ versus SFV/CHIKV IC_80_ (middle panel), and 3) PRNT_80_ versus CHIKV-luc NT_80_ (right panel). The correlation coefficient (r), number of samples measured by both assays (n), and statistical significance (p) are listed within each panel.

Thirty-three of the 146 samples were determined to be negative by at least one of the three assays. Twenty-seven of the 33 (82%) were negative for all 3 assays. For the six discrepancies between assays, the titer was close to the positivity cutoff (near the starting dilution of the assay). The number of samples in the three analyses ranged from 113 to 115. Using data from the VRC311 trial, a strong and statistically significant correlation was observed between results from the PRNT_80_ and SFV/CHIKV IC_80_ methods (r = 0.88, n = 113) and from the CHIKV-luc NT_80_ and SFV/CHIKV IC_80_ methods (r = 0.94, n = 113). There was also a strong and statistically significant correlation between the CHIKV-luc NT_80_ assay and PRNT_80_ (r = 0.89, n = 115). Overall, a strong correlation was observed for each of the 3 pairs of assays analyzed and the median bias between each pair was 15% or less. A summary of the correlation and bias of the CHIKV neutralization assays for samples with measurable titer between each pair analyzed is shown in Fig 5 and Table 2.

**Table 2:** Comparison of CHIKV-specific antibody titers obtained from PRNT_80_, SFV/CHIK OPY1-GFP IC_80_, and CHIKV-luc NT_80_ assay methods.

Assay Comparison	Measurable samples (n)	Correlation coefficient (r)	Median bias (95% CI)
PRNT_80_ vs. SFV/CHIKV IC_80_	113	0.88	−3% (−10%, 4%)
CHIKV-luc NT_80_ vs. SFV/CHIKV IC_80_	113	0.94	15% (1%, 24%)
PRNT_80_ vs. CHIKV-luc NT_80_	115	0.89	8% (−6%, 30%)

### 3.4. CHIKV-luc NT_80_ vs NT_50_

**Fig 6:**
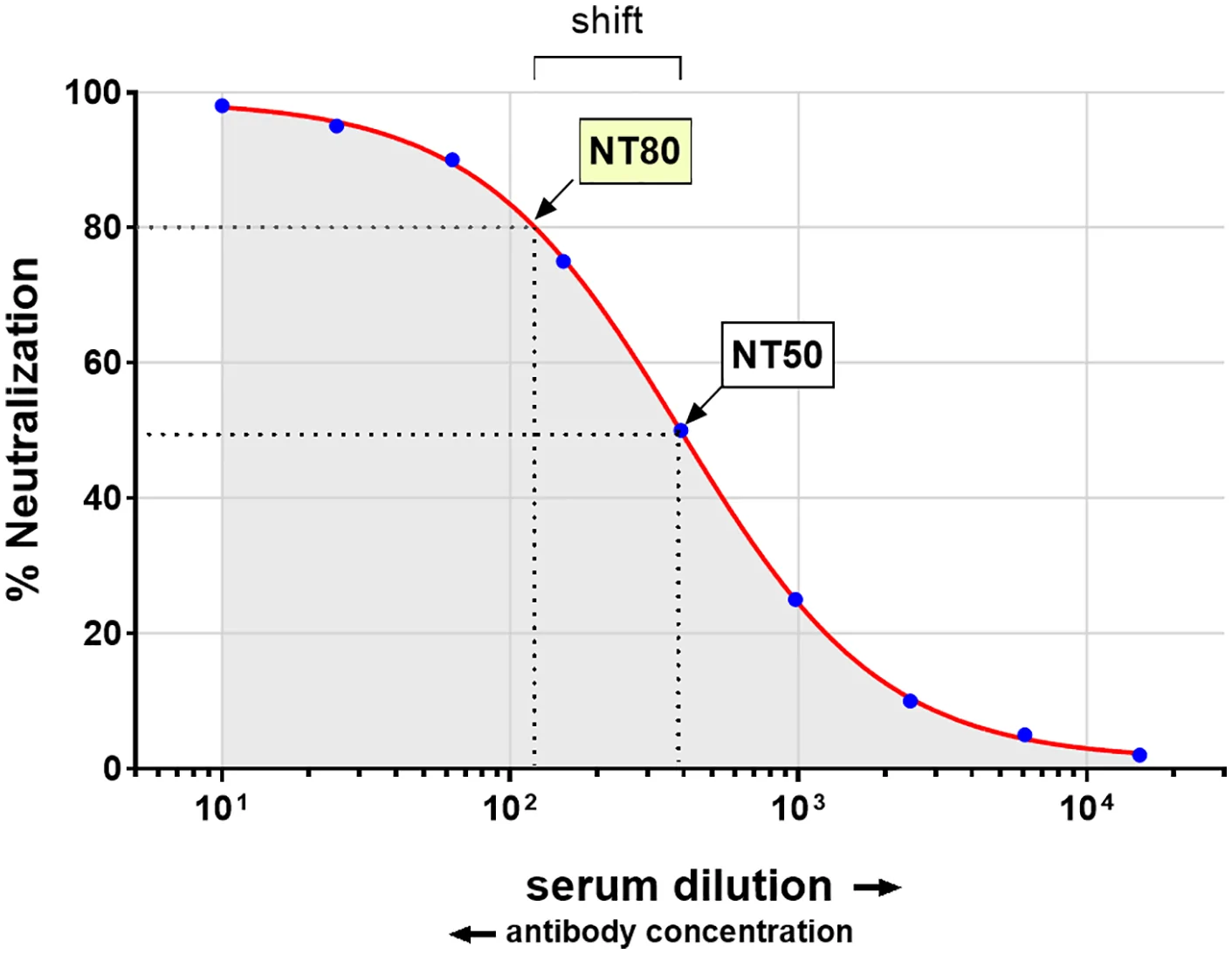
Neutralization curve. The neutralization curve shows percent virus neutralization versus serum dilution. NT_50_ and NT_80_ titers are marked on the curve to demonstrate the shift in antibody concentration required to achieve 80% vs 50% virus neutralization (inhibition of infection). The curve shows that higher antibody concentration is required to achieve 80% neutralization compared to 50%.

The neutralization titer was determined by calculating the percentage of virus neutralized after treatment with serum or plasma containing virus-specific neutralizing antibodies. In the context of CHIKV neutralization assays, 50% neutralization titer is commonly reported, however, 80% neutralization titers are sometimes used. These values are calculated from the sigmoid dose-response curve, or more appropriately the virus neutralization curve, representing the relationship between antibody concentration and the level of virus neutralization achieved. Plotting the log antibody concentration (serum dilution) on the x-axis and percentage of virus neutralization on the y-axis generates a sigmoidal logistics curve representing the typical response pattern of antibody neutralization, the neutralization curve (Fig 6). From the fitted curve, the antibody concentration or dilution that corresponds to 50% and 80% can be estimated (dotted lines). The curve shows that a higher antibody concentration (lower dilution) is needed to achieve an NT_80_ compared to NT_50_.

**Fig 7:**
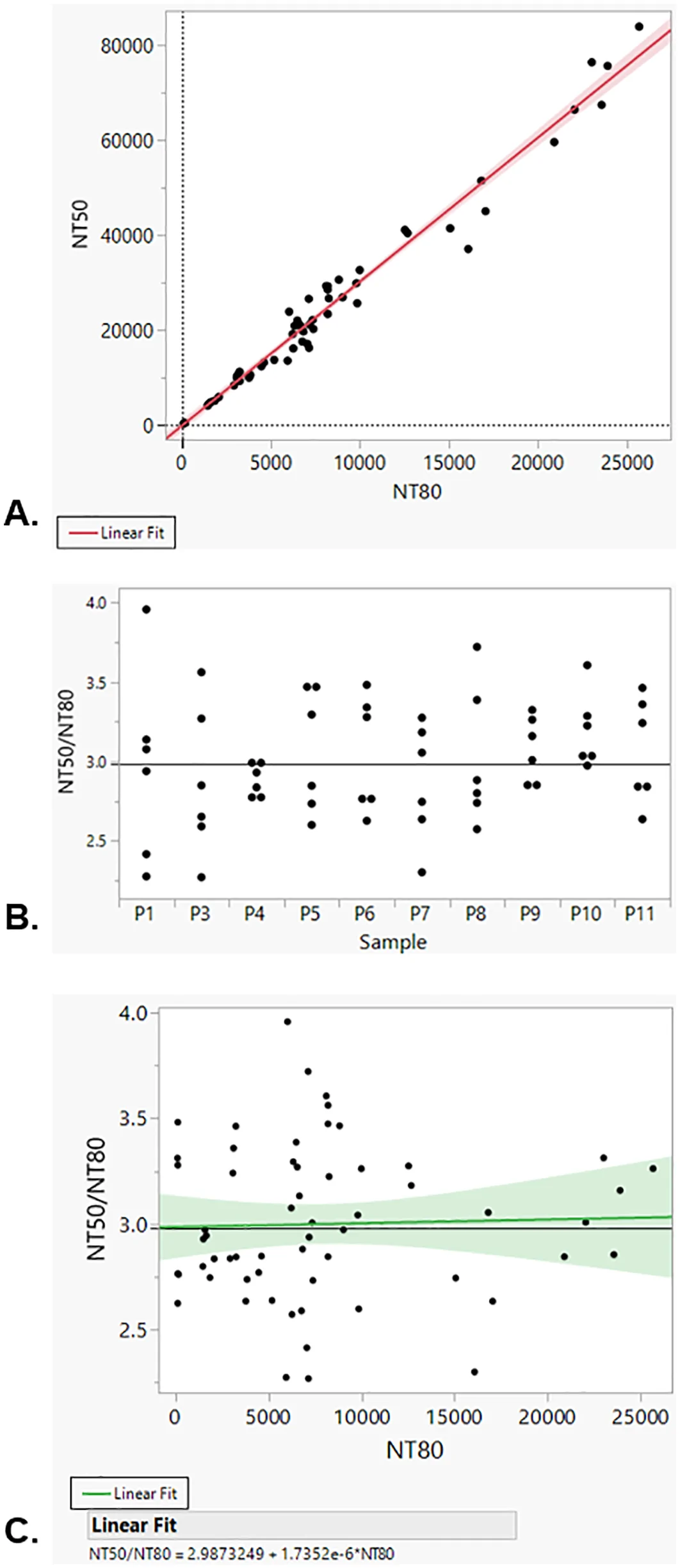
Linear fit of NT_50_ versus NT_80_, one-way analysis of variance, and bivariate fit. A. Linear fit of NT_50_ versus NT_80_ shows the linear relationship of NT_50_ and NT_80_ data with slope (red line) and the ratio of the best fit indicating a 3.03- fold difference. B. One-way analysis of NT_50_ and NT_80_ data shows the NT_50_/NT_80_ ratio for each sample (dots) in relation to the overall geometric mean ratio of 2.98 (black line). C. Bivariate fit of NT_50_/NT_80_ by NT_80_ shows the ratio stays the same over the NT_80_ range. The black line is the arithmetic mean and the green line shows that the slope CI overlaps 0, so no effect.

Linear regression and interpolation were used to calculate a more precise NT_50_ and NT_80_ titer. The fold difference between the NT_50_ and NT_80_ for a given sample is a ratio of [NT_50_/NT_80_]. The difference between NT_50_ and NT_80_ titer values can vary from sample to sample, but overall, it is approximately 3-fold. A linear fit helps visualize the relationship between NT_50_ and NT_80_ titer values as shown in Fig 7A. The linear fit of NT_50_ versus NT_80_ for samples shows the linear relationship between NT_50_ and NT_80_ data with R^2^ = 0.975, root mean square error = 3225.7, and slope = 3.031 [90% CI 2.906, 3.156], indicating a 3.03-fold difference. One-way analysis of variance (ANOVA) of the data shows the NT_50_/NT_80_ ratio (fold-difference) for replicate testing of each sample (P1, P3, P4, etc.) in relation to the geometric mean ratio of 2.98 (Fig 7B).

Variability is expected to increase as the neutralizing titer gets higher in value since more dilutions are needed to calculate higher titers (more dilutions can lead to more error). Therefore, variability may be worse for NT_50_ titer values, as they are 3-fold higher compared to NT_80_. Log transforming into regression analysis helps normalize the data, diminishing the variability observed linearly, making it more appropriate for statistical comparisons. The bivariate fit shows a trend of increasing variability for NT_50_ as the values increase; however, the slope is not statistically significant (p = 0.81); this demonstrates that ratios are consistent across the range and variability is associated with assay factors, and that NT_50_ and NT_80_ are not disparate measures (Fig 7C).

CHIKV-luc neutralization assay precision was determined using the 10 serum samples with detectable neutralization using both NT_50_ and NT_80_ endpoints (log-transformed), with overall assay precision and precision of each sample determined as %CV using the log-transformed variance. Assay precision for calculated IU/mL for both endpoints and showing repeatability and intermediate precision CVs are lower for NT_80_ (Table 3). The 10 serum samples (P1-P11) were tested in duplicate on 3 separate days (n = 6 per sample) and NT_50_ and NT_80_ titers were calculated (S1 Table). The results were then converted to U/mL relative to P1. The P1 sample corresponded to 1502/19 (the first WHO International Standard) and assigned a potency of 1000 IU/mL in agreement with study participants. The unitage was closely aligned with the overall mean potency across assay types. The CVs show that the variability of IU/mL is slightly lower for NT_80_.

**Table 3:** CHIKV-luc assay overall precision.

Endpoint	Random Effect	Precision	Variance	%CV	Standard Error	% of Total
Log[NT_50_ IU/mL]	Experimental	Day	0.011	10.7%	0.013	28.149
	Residual	Repeatability	0.029	17.2%	0.006	71.851
	Total	Intermediate Precision	0.040	20.3%	0.014	100.000
Log[NT_80_ IU/mL]	Experimental	Day	0.006	7.8%	0.007	25.090
	Residual	Repeatability	0.018	13.5%	0.004	74.910
	Total	Intermediate Precision	0.024	15.7%	0.008	100.000

### 3.5. CHIKV-luc NT_80_ assay validation

Validation results confirmed adequate performance of the assay by meeting the predefined acceptance criteria for each performance parameter (Table 4). Intra-assay precision had a CV of 13.6% and was required to have a CV < 25%. Inter-assay precision had a CV of 20.4% and was required to have a CV < 35%. Dilutional linearity had all 90% CI slopes within -1.3 and -0.7 and the R^2^ was 0.989, which was > 0.95. LOD was determined to be NT_80_ = 10 and LLOQ was determined to be NT_80_ > 15. Testing of robustness including freeze/thaw, sample stability, and incubation ranges for the neutralization step, overnight infection, and addition of Steady Glo reagent all showed 90% CI of ratios to be within 0.7 and 1.4.

**Table 4:** Summary of CHIKV-luc NT_80_ assay validation parameters, acceptance criteria, and results.

Parameter	Acceptance criteria	Results	Evaluation
**Intra-Assay Precision**	Overall intra-assay precision CV ≤ 25%	Overall intra-assay precision CV = 13.6%	Pass
**Inter-Assay Precision**	Overall inter-assay precision CV ≤ 35%	Overall inter-assay precision CV = 20.4%	Pass
**Dilutional Linearity**	90% CI of the slope of 1^st^ order linear regression analysis of log transformed titers between -1.3 and -0.7, and R^2^ > 0.95	All 90% CIs of slopes were within -1.3 and -0.7 Minimal R^2^ = 0.989 > 0.95	Pass
**LOD/LLOQ**	LOD was the lowest titer in which 95% of the tests resulted in a reportable titer >10 LLOQ was the lowest titer that met acceptance criteria for both intermediate precision and linearity and was at or above the LOD	100% of negative samples yielded titers <10. 100% of positive samples yielded titers >10 For samples with NT_80_ > 15, max CV was 31.2% (<35%) and passed linearity	Pass
**Robustness**			
Freeze/Thaw	90% CI of the ratio of each test condition to the reference condition within 0.7 to 1.4	All 90% CI of ratios were within 0.7 and 1.4	Pass
Sample Stability			Pass
Incubation Range - Neutralization			Pass
Incubation Range – Overnight Infection			Pass
Incubation Range – Steady-glo			Pass
**Selectivity**			
Hemolyzed Serum	90% CI of the ratio of each test condition to the reference condition within 0.7 to 1.4	All 90% CI of ratios were within 0.7 and 1.4	Pass
Lipemic Serum			Pass
Plasma			Pass

### 3.6. CHIKV-luc NT_80_ assay application

The WHO expert committee on biological standardization and Paul-Ehrlich-Institut (PEI) coordinated a collaborative study to evaluate an International Standard for antibodies to CHIKV [29]. In brief, the potency of the candidate International Standard, related reference serum, clinical and control samples were tested by a range of virus neutralization and immunoassays in laboratories from 26 different countries. Sixteen laboratories used their own in-house developed virus neutralization assays to test the panel of eleven total serum samples provided by PEI. Inter- and intra-assay precision was evaluated by testing the serum panel on three separate days using fresh dilutions of virus in each assay run. Raw data was provided to PEI, and a 4-parametric dose-response model was used to estimate 50% neutralization titer (PRNT_50_, NT_50_, or EC_50_). The combined mean titers calculated for each participating virus neutralizing assay including the CHIKV-luc NT_80_ assay consistently detected all CHIKV positive samples in the panel and the mean titers were all within 2 log_10_ of the candidate International Standard and related reference serum. Harmonization was also achieved when the titer data from the assay was expressed relative to the International Standard. Results from the study established the first International Standard for anti-CHIKV neutralizing antibodies, (code number 1502/19), with an assigned unitage of 1,000 International Units (IU) per mL (IU/mL).

The CHIKV-luc NT_80_ assay was used to test more than 25,000 serum samples collected across multiple timepoints in seven clinical trials evaluating the CHIKV VLP vaccine, five of which used the assay to support immunogenicity endpoints (Table 5).

**Table 5:** Clinical trials using the CHIKV-luc NT_80_ assay.

Clinical trial	ID	Phase	# participants	# timepoints	Samples tested^c^	Reference
NCT01489358	VRC311^a^	1	25	5	146	Chang 2014
NCT02562482	VRC704^b^	2	400	11	4,400	Chen 2020
NCT03483961	PXVX-CV-317–001	2	445	9^d^	4,405	Bennett 2022
NCT03992872	EBSI-CV-317–002	2	60	6	360	Hamer 2025
NCT05065983	EBSI-CV-317–010	2	25	5	125	n/a
NCT05072080	EBSI-CV-317–004	3	3258	5	16,290	Richardson 2025
NCT05349617	EBSI-CV-317–005	3	413	4	1,652	Tindale 2025
Total = 27,232						

^a^Retrospective sample testing

^b^Samples were tested and the data published, but did not directly support VRC704 endpoints

^c^Approximate number of samples tested based on number of participants and timepoints, not accounting for early withdrawals.

^d^The majority of the dose and schedule groups were tested at 9 timepoints, but groups ranged between 4 to 12 timepoints.

## 4. Discussion

The CHIKV-luc NT_80_ assay was developed to provide greater efficiency, reduced complexity, higher throughput, and enhanced scalability, offering a transformative advance over historically accepted assays and that overcome the limitations of PRNTs. Automation and electronic systems established a streamlined workflow with precision and consistency, making it well-suited for large-scale studies. The assay was also shown to be comparable to other neutralization assays, including the PRNT. In a prospective cohort field study by Yoon *et al*. [9], a baseline CHIKV PRNT_80_ titer ≥10, was associated with 100% protection from symptomatic CHIKV infection (95% CI: 46.1, 100.0). When comparing the CHIKV-luc NT_80_ and the PRNT_80_ assays, this corresponded to an NT_80_ titer of 35. The assay was successfully validated, demonstrating robust performance across all predefined analytical parameters. The findings of this study demonstrate that the CHIKV-luc NT_80_ assay provides a precise and quantitative measure of neutralizing activity, representing a robust and reliable alternative approach for assessing CHIKV neutralizing antibodies that correlates well with PRNT.

Unlike the commonly used NT_50_, this assay reports NT_80_ titers, a more stringent threshold chosen based on evidence that PRNT_80_ titers ≥10 correlate with protection after natural infection [9]. The fundamental difference between 50% and 80% neutralization titers lies in the percentage of virus inhibition measured. On average, CHIKV NT_80_ titers are approximately 3-fold lower than NT_50_ values and remain consistent across high and low titer ranges, as shown in this study. Although NT_80_ values appear numerically lower than NT_50,_ they represent higher neutralizing activity. Most assays report NT_50_ because it is historically well-established, offers greater sensitivity for detecting lower antibody levels, and is more practical for high-throughput settings. NT_50_ also facilitates cross-study comparison given its widespread use. However, NT_80_ provides a more stringent measure of neutralization, requiring the neutralization of a greater fraction of the virus in the assay, which reduces the likelihood of detecting low-level responses that may not confer meaningful protection. This stricter threshold was prioritized in the CHIKV VLP vaccine program to strengthen the rigor and clinical relevance of immunogenicity assessments.

Neutralization assays lack standardization across studies, complicating direct titer comparisons between trials and when using different platforms, such as PRNT, potentially misleading. Assay-specific factors, including viral strain, cell type, detection method, and endpoint definition, may influence the resulting titers. The CHIKV-luc NT_80_ assay, while conservative, offers clear advantages in throughput, reproducibility, and automation, and has been validated against the gold-standard PRNT. Consistent use across all CHIKV VLP vaccine phase 2 and 3 trials over 25,000 samples has enabled robust internal immunogenicity comparisons.

As CHIKV continues to spread globally, effective control efforts require fast, reliable, and scalable assays to accelerate vaccine development, outbreak preparedness, and data harmonization. The validated CHIKV-luc NT_80_ assay provides a sensitive and reproducible platform for measuring neutralizing antibodies. Using different lineages for the CHIKV-luc NT_80_ assay and the CHIKV VLP vaccine enables measurement of heterotypic neutralizing responses, reducing strain-specific bias and better reflecting real-world exposure to diverse circulating viruses, supporting the assay’s value in assessing broad, cross-protective immunity.

While the CHIKV-luc NT_80_ assay offers advantages in throughput, reproducibility, and stringency, certain considerations remain. Differences in viral strain, cell type, and endpoint definition compared to traditional PRNTs can influence results, and the lack of standardization across platforms complicates direct comparisons. Differences in viral strain, cell type, and endpoint definition relative to PRNTs, along with lack of cross-platform standardization, can affect comparability. The assay also requires BSL-2 conditions, cell culture infrastructure, specialized reagents, and luminescence-based detection, which may limit implementation outside laboratories with established virology capacity. While well suited for vaccine trials, assay harmonization, and reference laboratories, these requirements may constrain its use for routine serological surveillance in resource limited settings. Nonetheless, it could support surveillance through deployment in centralized or regional laboratories, providing high quality neutralization data to complement broader seroepidemiology.

Because the three neutralization platforms differ in sensitivity, dynamic range, and signal characteristics, each assay was performed using its validated dilution scheme to optimize curve quality and reproducibility. The CHIKV‑luc NT_80_ assay supports a broad linear range with a 1:10 starting dilution, whereas the PRNT requires a lower starting dilution to maintain plaque counts, and the GFP assay uses a 1:3 dilution to preserve signal above background. Although dilution intervals differed, titers were derived by nonlinear regression of the full neutralization curve rather than discrete endpoints, minimizing the impact of dilution spacing on titer estimation. Consistent with this, correlation analyses demonstrated strong agreement between assays with no evidence of proportional bias. While harmonized dilution schemes may suit assays with similar performance characteristics, enforcing a uniform format across platforms with distinct dynamic ranges would compromise performance. Using assay‑appropriate dilution schemes therefore supports accurate titer estimation while preserving valid inter‑assay comparisons.

Future directions include incorporating the WHO International Standard as a positive control in routine testing for improved assay comparability and facilitated harmonization. Continued efforts to standardize CHIKV neutralization assays and define clinically meaningful thresholds of protection will be critical for advancing vaccine development and regulatory alignment. Moreover, expanding the use of the CHIKV-luc NT_80_ assay to assess emerging CHIKV strains and vaccine candidates will help maintain its relevance in an evolving epidemiological landscape.

The CHIKV-luc NT_80_ assay played a pivotal role in supporting the licensure pathway for CHIKV VLP vaccine, reliably assessing vaccine-induced immunogenicity across three phase 2 trials and two phase 3 trials. Beyond its application in clinical studies, the assay was utilized in passive transfer experiments in NHPs to help define a surrogate threshold of protection [36]. Its successful development, validation, and integration across preclinical research, clinical trials, international standardization efforts, and regulatory submissions enabled a seamless transition from early assay development to vaccine licensure. This work underscores the value of scalable, quantitative platforms in accelerating vaccine development pipelines. As emerging pathogens continue to challenge global health systems, the CHIKV-luc NT_80_ assay serves as a model for how reporter virus-based technologies can streamline immunogenicity assessment, harmonize data across studies, and support rapid vaccine deployment. The success of this assay within the CHIKV VLP vaccine program provides a blueprint for adapting similar approaches to other pathogens, advancing assay innovation for outbreak response and preparedness.

## Supporting information

S1 Table CHIKV-luc NT assay precision. (DOCX)

S2 Table Assay comparison dataset. (DOCX)
