# Supplementary material for: Luciferase unleashed: Lighting the path to vaccine approval using a novel high-throughput chikungunya virus neutralization assay
Source: PLoS Negl Trop Dis. 2026 Aug 3;20(8):e0014610. doi: 10.1371/journal.pntd.0014610 (PMC13506084; doi:10.1371/journal.pntd.0014610)
Supplement: Supplementary file 1 [file pntd.0014610.s001.docx]

SUPPORTING INFORMATION

Table S 1. CHIKV-luc NT assay precision

| Endpoint | NT_50_ | | NT_80_ | |
| --- | --- | --- | --- | --- |
| Sample | U/mL | %CV | U/mL | %CV |
| P1 | 1000 | 20.1 | 1000 | 8.1 |
| P3 | 940 | 30.6 | 969 | 21.5 |
| P4 | 253 | 12.5 | 257 | 13.5 |
| P5 | 1296 | 16.8 | 1240 | 15.3 |
| P6 | 19 | 17.3 | 18 | 16.6 |
| P7 | 2241 | 11.3 | 2297 | 13.9 |
| P8 | 897 | 36.3 | 876 | 25.8 |
| P9 | 3748 | 12.3 | 3563 | 7.1 |
| P10 | 1460 | 13.4 | 1340 | 12.0 |
| P11 | 518 | 10.4 | 496 | 8.7 |

CV = coefficient of variation; NT = neutralization titer.

Note: Samples P1 and P3 are replicate samples and were blinded in the study. Values for P2 (negative control) are not shown.
