# Supplementary material for: Luciferase unleashed: Lighting the path to vaccine approval using a novel high-throughput chikungunya virus neutralization assay
Source: PLoS Negl Trop Dis. 2026 Aug 3;20(8):e0014610. doi: 10.1371/journal.pntd.0014610 (PMC13506084; doi:10.1371/journal.pntd.0014610)
Supplement: Supplementary file 2 [file pntd.0014610.s002.docx]

Table S 2. Assay comparison dataset

| Sample # | Participant # | Dose | Visit # | Timepoint (week) | IC_80_ | PRNT_80_ | NT_80_ |
| --- | --- | --- | --- | --- | --- | --- | --- |
| 1 | 1 | 10mcg | 2 | 0 | < 50 | < 10 | <10 |
| 2 |  | 10mcg | 3 | 4 | < 50 | 10.0 | 13.6 |
| 3 |  | 10mcg | 4 | 8 | 703.4 | 920.3 | 1219.4 |
| 4 |  | 10mcg | 5 | 20 | 323.5 | 343.1 | 483.6 |
| 5 |  | 10mcg | 6 | 24 | 1638.5 | 1138.4 | 2325.0 |
| 6 |  | 10mcg | 7 | 44 | 225.8 | 320.0 | 511.0 |
| 7 | 2 | 10mcg | 2 | 0 | < 50 | < 10 | <10 |
| 8 |  | 10mcg | 3 | 4 | 230.8 | 109.3 | 211.7 |
| 9 |  | 10mcg | 4 | 8 | 3163 | 3194.9 | 2815.1 |
| 10 |  | 10mcg | 5 | 20 | 395.3 | 367.3 | 486.5 |
| 11 |  | 10mcg | 6 | 24 | 1202 | 1398.1 | 2756.3 |
| 12 |  | 10mcg | 7 | 44 | 237.1 | 597 | 487.0 |
| 13 | 3 | 10mcg | 2 | 0 | < 50 | < 10 | <10 |
| 14 |  | 10mcg | 3 | 4 | 753.7 | 520.1 | 337.1 |
| 15 |  | 10mcg | 4 | 8 | 544.5 | 871 | 499.9 |
| 16 |  | 10mcg | 5 | 20 | 100.4 | 68.3 | 65.3 |
| 17 |  | 10mcg | 6 | 24 | 2332 | 2501 | 3642.0 |
| 18 |  | 10mcg | 7 | 44 | 110.1 | 311.2 | 203.0 |
| 19 | 4 | 10mcg | 2 | 0 | < 50 | < 10 | <10 |
| 20 |  | 10mcg | 3 | 4 | 91.71 | 226.3 | 202.5 |
| 21 |  | 10mcg | 4 | 8 | 442.9 | 749.8 | 596.8 |
| 22 |  | 10mcg | 5 | 20 | 260 | 435.4 | 199.7 |
| 23 |  | 10mcg | 6 | 24 | 35371 | 21761.8 | 42897.0 |
| 24 |  | 10mcg | 7 | 44 | 5074 | 20480 | 14512.0 |
| 25 | 5 | 10mcg | 2 | 0 | < 50 | < 10 | <10 |
| 26 |  | 10mcg | 3 | 4 | < 50 | < 10 | 13.4 |
| 27 |  | 10mcg | 4 | 8 | 1589 | 935.3 | 962.2 |
| 28 |  | 10mcg | 5 | 20 | 254.2 | 155.7 | 202.0 |
| 29 |  | 10mcg | 6 | 24 | 2488 | 1971.8 | 2169.0 |
| 30 |  | 10mcg | 7 | 44 | 310.8 | 309.1 | 679.0 |
| 31 | 6 | 20mcg | 2 | 0 | < 50 | < 10 | <10 |
| 32 |  | 20mcg | 3 | 4 | < 50 | < 10 | <10 |
| 33 |  | 20mcg | 4 | 8 | 630.6 | 583.5 | 864.8 |
| 34 |  | 20mcg | 5 | 20 | 220.2 | 175.3 | 308.2 |
| 35 |  | 20mcg | 6 | 24 | 2550 | 2323.4 | 2274.0 |
| 36 |  | 20mcg | 7 | 44 | 518 | 278.6 | 849.0 |
| 37 | 7 | 20mcg | 2 | 0 | < 50 | < 10 | 13.1 |
| 38 |  | 20mcg | 3 | 4 | 126 | 199.5 | 86.5 |
| 39 |  | 20mcg | 4 | 8 | 1924 | 1620 | 2651.2 |
| 40 |  | 20mcg | 5 | 20 | 502.5 | 259.9 | 599.0 |
| 41 |  | 20mcg | 6 | 24 | 10910 | 12033.9 | 12433.4 |
| 42 |  | 20mcg | 7 | 44 | 4901 | 4831.8 | 10303.3 |
| 43 | 8 | 20mcg | 2 | 0 | < 50 | < 10 | <10 |
| 44 |  | 20mcg | 3 | 4 | 384.3 | 422.3 | 1254 |
| 45 |  | 20mcg | 4 | 8 | 383.1 | 207.7 | 416.4 |
| 46 |  | 20mcg | 5 | 20 | 112.8 | 35.8 | 102.5 |
| 47 |  | 20mcg | 6 | 24 | 418.6 | 222.4 | 708.2 |
| 48 |  | 20mcg | 7 | 44 | 280.5 | 370.1 | 520.8 |
| 49 | 9 | 20mcg | 2 | 0 | < 50 | < 10 | <10 |
| 50 |  | 20mcg | 3 | 4 | 1381 | 1499.8 | 972.1 |
| 51 |  | 20mcg | 4 | 8 | 1236 | 1262.4 | 2009.6 |
| 52 |  | 20mcg | 5 | 20 | 215.2 | 422.4 | 265.3 |
| 53 |  | 20mcg | 6 | 24 | 2305 | 2319.1 | 1504.1 |
| 54 |  | 20mcg | 7 | 44 | 765.7 | 1003.8 | 909.5 |
| 55 | 10 | 20mcg | 2 | 0 | < 50 | < 10 | <10 |
| 56 |  | 20mcg | 3 | 4 | < 50 | 10 | <10 |
| 57 |  | 20mcg | 4 | 8 | 302.9 | 367.6 | 172.9 |
| 58 |  | 20mcg | 5 | 20 | 131.5 | 104.1 | 90.2 |
| 59 |  | 20mcg | 6 | 24 | 872.3 | 1527.9 | 791.6 |
| 60 |  | 20mcg | 7 | 44 | 132 | 174.1 | 141.5 |
| 61 | 11 | 20mcg | 2 | 0 | < 50 | < 10 | <10 |
| 62 |  | 20mcg | 3 | 4 | 135.3 | 199.5 | 161.9 |
| 63 |  | 20mcg | 4 | 8 | 1166 | 1318.3 | 1483.0 |
| 64 |  | 20mcg | 5 | 20 | 128.5 | 190.5 | 81.9 |
| 65 |  | 20mcg | 6 | 24 | 339.8 | 407.4 | 307.3 |
| 66 |  | 20mcg | 7 | 44 | 70.79 | 120.2 | 88.0 |
| 67 | 12 | 20mcg | 2 | 0 | < 50 | < 10 | <10 |
| 68 |  | 20mcg | 3 | 4 | 189.8 | 206.3 | 555.5 |
| 69 |  | 20mcg | 4 | 8 | 319.1 | 787.9 | 463.1 |
| 70 |  | 20mcg | 5 | 20 | 89.8 | 99.5 | 128.1 |
| 71 |  | 20mcg | 6 | 24 | 1143 | 2890.0 | 1756.8 |
| 72 |  | 20mcg | 7 | 44 | 74.57 | 76.4 | 133.5 |
| 73 | 13 | 20mcg | 2 | 0 | < 50 | < 10 | <10 |
| 74 |  | 20mcg | 3 | 4 | 75.51 | 82.3 | 95.9 |
| 75 |  | 20mcg | 4 | 8 | 982.1 | 1324.9 | 2095.4 |
| 76 |  | 20mcg | 5 | 20 | 162.1 | 169.8 | 161.1 |
| 77 |  | 20mcg | 6 | 24 | 2646 | 1575.8 | 1564.8 |
| 78 |  | 20mcg | 7 | 44 | 327.7 | 375.5 | 443.0 |
| 79 | 14 | 20mcg | 2 | 0 | < 50 | < 10 | <10 |
| 80 |  | 20mcg | 3 | 4 | < 50 | < 10 | < 10 |
| 81 |  | 20mcg | 4 | 8 | 560.2 | 197.8 | 236.3 |
| 82 |  | 20mcg | 5 | 20 | 90.95 | 69.6 | 103.5 |
| 83 |  | 20mcg | 6 | 24 | 910.4 | 685.8 | 1034.2 |
| 84 |  | 20mcg | 7 | 44 | 100.1 | 214.8 | 132.1 |
| 85 | 15 | 20mcg | 2 | 0 | < 50 | < 10 | <10 |
| 86 |  | 20mcg | 3 | 4 | 283.1 | 344.8 | 271.6 |
| 87 |  | 20mcg | 4 | 8 | 734 | 861.3 | 797.2 |
| 88 |  | 20mcg | 5 | 20 | 340.2 | 326.9 | 308.3 |
| 89 |  | 20mcg | 6 | 24 | 815.1 | 806.6 | 532.7 |
| 90 |  | 20mcg | 7 | 44 | 179.2 | 377.9 | 279.7 |
| 91 | 16 | 40mcg | 2 | 0 | < 50 | < 10 | <10 |
| 92 |  | 40mcg | 3 | 4 | 247.8 | 357.3 | 278.3 |
| 93 |  | 40mcg | 4 | 8 | 1549 | 1059.7 | 1872.6 |
| 94 |  | 40mcg | 5 | 20 | 431.5 | 416.9 | 517.6 |
| 95 |  | 40mcg | 6 | 24 | 962.8 | 1940.9 | 1727.0 |
| 96 |  | 40mcg | 7 | 44 | 651.6 | 520.0 | 670.5 |
| 97 | 17 | 40mcg | 2 | 0 | < 50 | < 10 | 12.4 |
| 98 |  | 40mcg | 3 | 4 | < 50 | 116.9 | 251.4 |
| 99 |  | 40mcg | 4 | 8 | 1901 | 1570.0 | 1920.5 |
| 100 |  | 40mcg | 5 | 20 | 358.9 | 164.2 | 363.7 |
| 101 |  | 40mcg | 6 | 24 | 789.85 | 769.9 | 1254.0 |
| 102 |  | 40mcg | 7 | 44 | 207.2 | 266.6 | 239.0 |
| 103 | 18 | 40mcg | 2 | 0 | < 50 | < 10 | <10 |
| 104 |  | 40mcg | 3 | 4 | 621.7 | 349.0 | 563.3 |
| 105 |  | 40mcg | 4 | 8 | 2670 | 422.7 | 2124.2 |
| 106 |  | 40mcg | 5 | 20 | 591 | 350.9 | 493.0 |
| 107 |  | 40mcg | 6 | 24 | 3610 | 5286.1 | 3660.4 |
| 108 |  | 40mcg | 7 | 44 | 952.4 | 255.5 | 1356.0 |
| 109 | 19 | 40mcg | 2 | 0 | < 50 | < 10 | <10 |
| 110 |  | 40mcg | 3 | 4 | 1295.05 | 2768.4 | 2290.3 |
| 111 |  | 40mcg | 4 | 8 | 983.6 | 587.0 | 447.9 |
| 112 |  | 40mcg | 5 | 20 | 219.1 | 224.9 | 299.3 |
| 113 |  | 40mcg | 6 | 24 | 120.3 | 316.4 | 207.0 |
| 114 |  | 40mcg | 7 | 44 | 120.4 | 158.0 | 132.1 |
| 115 | 20 | 40mcg | 2 | 0 | < 50 | < 10 | <10 |
| 116 |  | 40mcg | 3 | 4 | 59.68 | 157.8 | 92.3 |
| 117 |  | 40mcg | 4 | 8 | 8926 | 10379.6 | 6498.4 |
| 118 |  | 40mcg | 5 | 20 | 1067 | 234.3 | 914.8 |
| 119 |  | 40mcg | 6 | 24 | 2357 | 2356.6 | 3640.0 |
| 120 |  | 40mcg | 7 | 44 | 448.5 | 185.9 | 388.4 |
| 121 | 21 | 40mcg | 2 | 0 | < 50 | < 10 | <10 |
| 122 |  | 40mcg | 3 | 4 | 548.5 | 269.0 | 809.2 |
| 123 |  | 40mcg | 4 | 8 | 3572 | 3327.1 | 4708.0 |
| 124 |  | 40mcg | 5 | 20 | 486 | 422.2 | 375.8 |
| 125 |  | 40mcg | 6 | 24 | 9286 | 12730.6 | 12647.5 |
| 126 | 22 | 40mcg | 2 | 0 | < 50 | < 10 | <10 |
| 127 |  | 40mcg | 3 | 4 | 195.7 | 213.2 | 174.8 |
| 128 |  | 40mcg | 4 | 8 | 4575 | 2937.2 | 3185.2 |
| 129 |  | 40mcg | 5 | 20 | 867.5 | 691.3 | 604.3 |
| 130 |  | 40mcg | 6 | 24 | 2976 | 5582.9 | 3566.0 |
| 131 |  | 40mcg | 7 | 44 | 1494 | 1292.6 | 1731.7 |
| 132 | 23 | 40mcg | 2 | 0 | < 50 | < 10 | <10 |
| 133 |  | 40mcg | 3 | 4 | < 50 | < 10 | < 10 |
| 134 |  | 40mcg | 4 | 8 | 1800 | 1923.6 | 1040.0 |
| 135 |  | 40mcg | 5 | 20 | 208 | 53.4 | 146.5 |
| 136 |  | 40mcg | 6 | 24 | 2213 | 2786.5 | 3015.1 |
| 137 |  | 40mcg | 7 | 44 | 388.1 | 561.7 | 691.8 |
| 138 | 24 | 40mcg | 2 | 0 | < 50 | < 10 | <10 |
| 139 |  | 40mcg | 3 | 4 | < 50 | < 10 | < 10 |
| 140 |  | 40mcg | 4 | 8 | 1520 | 2355.4 | 1005.1 |
| 141 | 25 | 40mcg | 2 | 0 | < 50 | < 10 | <10 |
| 142 |  | 40mcg | 3 | 4 | 269.2 | 367.6 | 224.2 |
| 143 |  | 40mcg | 4 | 8 | 3144 | 1064.2 | 1838.9 |
| 144 |  | 40mcg | 5 | 20 | 663.9 | 212.8 | 338.7 |
| 145 |  | 40mcg | 6 | 24 | 2904 | 2521.7 | 2630.8 |
| 146 |  | 40mcg | 7 | 44 | 1643 | 1597.7 | 1671.1 |
